# Apparent Pacer Spikes in a Patient with Back and Chest Pain

**DOI:** 10.5811/cpcem.2019.1.41095

**Published:** 2019-02-26

**Authors:** Al Lulla, Richard T. Griffey

**Affiliations:** Washington University School of Medicine, Division of Emergency Medicine, St. Louis, Missouri

## CASE PRESENTATION

**Image 1:**
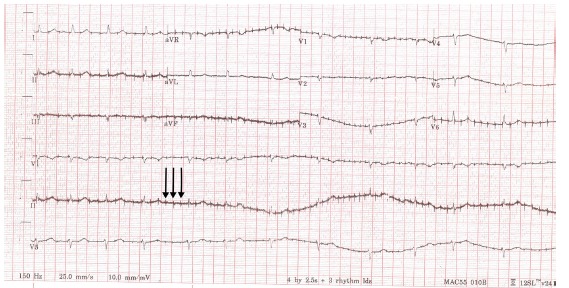
12-lead electrocardiogram demonstrates an irregular rhythm without p-wave morphologies and heart rate of 75, suggestive of rate-controlled atrial fibrillation. In addition, atypical, high-frequency electrical discharges (arrows) are seen irrespective of ventricular depolarization suggestive of aberrant pacemaker activity.

A 74-year-old female with a history of diabetes mellitus, hypertension, atrial fibrillation (on warfarin, diltiazem and metoprolol) presented with chest and back pain. A 12-lead electrocardiogram (ECG) was ordered at triage demonstrating possible aberrant pacemaker activity (Image 1).

## DIAGNOSIS

**Image 2:**
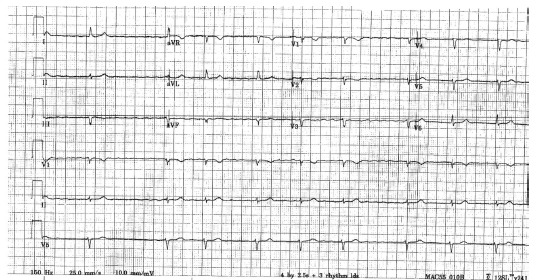
Repeat 12-lead electrocardiogram after removal of transcutaneous electrical nerve stimulation device demonstrates atrial fibrillation with slow ventricular response and resolution of electrical discharges.

Upon evaluation, the treatment team determined that the patient had no history of pacemaker placement. Physical examination revealed that the patient had an over-the-counter transcutaneous electrical nerve stimulation (TENS) unit adhered to her back to treat her back pain. Once the TENS device was removed, a repeat ECG demonstrated rate-controlled atrial fibrillation without any other electrical discharges (Image 2).

TENS units are external electrostimulators that produce electrical current with variable frequency, amplitude and duration, delivered through skin electrodes.^1^ TENS units are sometimes used in the treatment of post-operative and chronic pain, and are theorized to work by altering electrical signals involved with the perception of pain.^2^ Until recently, TENS units were only available through pain clinics or specialty providers. These units are now widely available over the counter, with an increasing likelihood of use by patients visiting the emergency department (ED). Although case reports from over 20 years ago report electrical spikes on ECG caused by earlier TENS units, this phenomenon has not been reported in the ED or in the emergency medicine literature and these electrical artifacts have not been described in newer, commercially available over-the-counter units in use today.^3^

Artifactual ECG changes inconsistent with a patient’s presentation, particularly in patients with back pain, should prompt consideration by the emergency physician of the presence of a TENS unit causing artifacts.

**CPC-EM Capsule**

What do we already know about this clinical entity?

*Electrocardiogram (ECG) artifacts are well described phenomena attributable to various sources. While traditional Transcutaneous Electrical Nerve Stimulation (TENS) units have been known to cause ECG changes, ECG abnormalities due to over the counter TENS units in the emergency department (ED) setting have not been previously reported*.

What is the major impact of the image(s)?

TENS devices are a known pain treatment modality that are now increasingly available over the counter. These devices may induce artifactual changes on ECGs

How might this improve emergency medicine practice?

*Careful and thorough history and physical exam in the emergency department may prompt the emergency physician to the presence of a TENS device as the cause of electrocardiographic artifact*.

## References

[1] Patel SI, Souter MJ (2008). Equipment-related electrocardiographic artifacts: causes, characterstics, and correction. Anesthesiology.

[2] Sliwa J, Marinko M (1996). Transcutaenous electrical nerve stimulator-induced electrocardiogram artifact: a brief report. Am J Pysical Med Rehabil.

[3] Hauptman PJ, Raza M (1992). Electrocardiographic artifact with a transcutaneous electrical nerve stimulation unit. Int J Cardiol.

